# Spatially localized sparse approximations of deep features for breast mass characterization

**DOI:** 10.3934/mbe.2023706

**Published:** 2023-08-01

**Authors:** Chelsea Harris, Uchenna Okorie, Sokratis Makrogiannis

**Affiliations:** Division of Physics, Engineering, Mathematics, and Computer Science, Delaware State University, 1200 N DuPont Hwy, Dover, DE 19901, USA

**Keywords:** breast mass classification, sparse approximation, deep features, mammography, convolutional neural network, patch decomposition and reconstruction

## Abstract

We propose a deep feature-based sparse approximation classification technique for classification of breast masses into benign and malignant categories in film screen mammographs. This is a significant application as breast cancer is a leading cause of death in the modern world and improvements in diagnosis may help to decrease rates of mortality for large populations. While deep learning techniques have produced remarkable results in the field of computer-aided diagnosis of breast cancer, there are several aspects of this field that remain under-studied. In this work, we investigate the applicability of deep-feature-generated dictionaries to sparse approximation-based classification. To this end we construct dictionaries from deep features and compute sparse approximations of Regions Of Interest (ROIs) of breast masses for classification. Furthermore, we propose block and patch decomposition methods to construct overcomplete dictionaries suitable for sparse coding. The effectiveness of our deep feature spatially localized ensemble sparse analysis (DF-SLESA) technique is evaluated on a merged dataset of mass ROIs from the CBIS-DDSM and MIAS datasets. Experimental results indicate that dictionaries of deep features yield more discriminative sparse approximations of mass characteristics than dictionaries of imaging patterns and dictionaries learned by unsupervised machine learning techniques such as K-SVD. Of note is that the proposed block and patch decomposition strategies may help to simplify the sparse coding problem and to find tractable solutions. The proposed technique achieves competitive performances with state-of-the-art techniques for benign/malignant breast mass classification, using 10-fold cross-validation in merged datasets of film screen mammograms.

## 1. Introduction

Cancer is one of the leading causes of death worldwide [1]. Among women, breast cancer is the most commonly diagnosed type of cancer [2]. It is projected that about 12% of all women in the U.S. will be diagnosed with breast cancer in their lifetime [3,4]. Mammography is one of the main imaging modalities used initially to diagnose breast cancer and is a standard preventive measure [5]. However, mammography examination by radiologists with variable clinical experience and training, poses the issue of variability in radiologist interpretive performance [6]. Thus, an automated computer-aided diagnosis (CAD) system would be a useful assistive tool in modern medicine and a second opinion for medical professionals. Automated computer-aided diagnosis systems can ultimately improve diagnosis accuracy, and reduce the time and expenses of diagnostic workflows.

Two important stages of conventional image classification systems are feature extraction and implementation of a classifier. Feature extraction is a process by which image descriptors/features are found, ideally, features that have the most discriminative power. CAD systems have used handcrafted features, such as texture features and statistical features to train a classifier [7-10]. In [8], a statistical t-test feature extraction and feature quantity optimization through thresholding method was used to distinguish between benign and malignant tumors. The extracted image features were fed into a support vector machine (SVM) classifier to perform class assignment.

Another category of techniques leverages the inherent sparsity of signals in nature to produce signal representations suitable for coding, superresolution and classification [11-14]. These techniques aim to approximate test signals by linear combinations of column vectors (or atoms) chosen from dictionary matrices that minimize sparsity under residual approximation constraints. These matrices may be directly sampled from the training set, or learned from it, using dictionary learning approaches typically based on the K-SVD algorithm [11].

Furthermore, state-of-the-art machine learning systems, like deep neural networks, have shown considerable applicability in medical imaging classification tasks. An ideal dataset of medical images used in machine learning would have physician annotations and contain a sufficient number of data samples (i.e., millions of medical images) [15]. However, medical image datasets with these ideal components are not widely available yet. Considering the lack of sufficient data, several deep neural network classification approaches apply pretrained networks to medical imaging data [16, 17]. Convolutional neural networks (CNNs), have achieved impressive performance in medical image classification and recent work concentrates on mammography data [2, 18-26]. In [18], transfer learning and data augmentation was used to overcome the challenge of limited mammography data. They validated end-to-end CNN classification on a shallow CNN architecture and two early CNNs, AlexNet and Googlenet. The authors in [23] proposed deep learning lesion detection and CNN classification. They compared the performance of a basic CNN to a modified ResNet50 and InceptionResNetV2 architecture for breast lesion classification. Their experimental results showed that deep learning CAD systems achieved better accuracy performance than conventional systems such as SVM. In [20], transfer learning and fine-tuning was applied to the AlexNet and Googlenet architectures for breast mass classification. The authors observed that transfer learning outperformed a shallow CNN trained from scratch. The feature extraction capacity of CNNs, combined with a traditional classifier, is evaluated in [19]. The authors apply an ensemble classifier approach to classify benign and malignant mass ROIs from full field digital mammograms. The ensemble classifier averaged the output of an SVM classifier trained on CNN features and an SVM classifier trained on analytically extracted features. The ensemble classifier outperformed the individual SVM classifiers.

Despite the significant progress in this field, there is still a need for effective and interpretable classification models that may use the representation capacity of deep learning and be trainable on small-sized datasets. In this work, we leverage the strengths of inductive representation capacity of features computed by deep convolutional networks to form dictionaries for sparse approximation-based classification of breast masses in mammograms. An original contribution of this work is that it investigates the suitability of deep feature maps as dictionaries to be used for sparse approximations. In addition, the sparse coding module can be used for visualization and interpretability of deep learning. For example, we can display the atoms/training samples that best approximate an unknown sample. Furthermore, we develop and evaluate block and patch selection, reconstruction and decision fusion techniques to increase the number and diversity of dictionary atoms for sparse analysis. We apply our deep feature spatially localized sparse analysis method (DF-SLESA) on a merged dataset of breast mass regions of interest (ROIs) from mammograms for separation of benign and malignant masses. We compare this technique to fully-sparse-based classifiers, and to end-to-end CNN classification. Experimental results suggest that deep feature-based dictionaries yield more discriminant sparse approximations of mass characteristics than pixel intensity-based dictionaries and K-SVD learned dictionaries [11], and improve classification performance.

## 2. Methods

In this section, we first describe the CNN architectures that will be interfaced with the sparse approximation stage. We then detail our Deep Feature-Spatially Localized Ensemble Sparse Analysis (DF-SLESA) technique.

### 2.1. Convolutional neural network descriptions

#### 2.1.1. Googlenet

The Googlenet introduced a network architecture that utilizes the Inception module. The Googlenet made its debut as a submission in the ILSVRC14 competition and outperformed in accuracy performance over the revolutionary network, Alexnet, with 10 times fewer parameters. It was designed with practical use and computational expense in mind. This network first employs two convolutional layers, each followed by max pooling. Next, the architecture includes 3 stages of Inception modules each followed by max pooling. The final Inception module is followed by an average pooling layer. Googlenet introduces a 1 × 1 convolutional kernel that computes reductions before convolutions in the Inception module to reduce computational expense. Convolution kernels of size 1 × 1, 3 × 3, and 5 × 5 are employed within an Inception module and the outputs of all convolutions are concatenated to produce the final feature map. To tackle the gradient vanishing problem during training, auxiliary classifiers are added to intermediate layers. A linear layer with a softmax loss is used as the classifier [27].

#### 2.1.2. InceptionV3

InceptionV3 shares similarities with the Googlenet as it includes a sequence of convolutional layers followed by Inception modules and a linear softmax classifier. Some of the unique properties of the InceptionV3 network are the factorization into smaller convolutions using 3 × 3 kernels, asymmetric convolutions to reduce the number of parameters, and batch normalization in the fully connected layer of the auxiliary classifier. InceptionV3 produced the lowest Top-1 and Top-5 errors on the ImageNet dataset at the time it was published [28].

#### 2.1.3. ResNet architectures

In 2015, a new network architecture known as Residual Network or ResNet, emerged in deep learning and introduced residual connections, which were beneficial for training deeper networks. The main concept is that shortcut connections (or skip connections) can be added to a plain network to facilitate learning of the deeper layers. Skip connections essentially allow activations from a layer to be fed to a layer deeper in the network. The ResNet family of CNNs follows a structure of an initial convolution stage without skip connections, followed by multiple stages of convolutional layers with residual connections, average pooling, and ending with a fully connected layer. In [29], deeper ResNets such as ResNet50 and ResNet101 yielded significantly better classification accuracy than the baseline ResNet18 and ResNet34 models.

#### 2.1.4. DenseNet

Recent studies have shown that shorter connections between layers near the input and near the output produce greater training efficiency, accuracy improvement, and support increased network depth. The authors in [30] proposed a densely connected convolutional neural network, DenseNet, that has $\frac{L(L+1)}{2}$ direct connections, unlike traditional L-layer CNNs that have L connections. DenseNets use a simple layer connection where all layers are connected directly to each other. The feed forward nature is maintained by ensuring every layer receives additional inputs from previous layers and passes them on as feature maps. Features are combined through concatenation (unlike summation as in ResNets) before passing on to subsequent layers. An important difference between DenseNet and other networks is that DenseNet can have very narrow layers using a growth rate hyperparameter.

#### 2.1.5. InceptionResNetV2

The InceptionResNetV2 [31] network is an extension of the InceptionV3 network. InceptionResNetV2 incorporates residual connections into the Inception architecture. The experimental results presented in [31], show that the use of residual connections in the Inception architecture accelerates training. The InceptionResNetV2 architecture begins with a stem block that has a series of 3 × 3 and 1 × 1 convolutions of different strides, filter concatenation and max pooling. Subsequently, this design employs multiple InceptionResNet1-A modules followed by a reduction layer, and multiple InceptionResNet1-B modules also followed by a reduction layer. The network architecture ends with multiple InceptionResNet1-C modules, average pooling and a softmax layer for classification.

#### 2.1.6. Xception

The Xception (or Extreme Inception) network proposed in [32], applies depthwise separable convolution layers. The Xception network architecture has a three stage model that consists of an entry flow, middle flow and exit flow. In the entry flow, two initial convolution layers perform 3 × 3 convolutions, each followed by a ReLU layer. Subsequently, three separable convolution blocks follow and the entry flow outputs 728 feature maps of size 19 × 19. The middle flow has one separable convolutional block that is repeated 8 times. The exit flow takes as input the output of the middle flow, performs additional separable convolutions, global average pooling, and lastly logistic regression. The depthwise separable convolutional layers function like Inception modules. Experimental results reported in [32] show that when residual connections are added to the Xception architecture there is a significant boost in accuracy.

### 2.2. Deep feature-spatially localized ensemble sparse analysis

We combine localized sparse approximations and the feature extraction capabilities of convolutional neural networks to classify breast masses into malignant or benign states. The DF-SLESA method workflow has three major stages, namely, deep feature extraction, deep dictionary construction and ensemble classification and decision making [33]. Further details of the DF-SLESA method and its main stages are given in the following sections.

#### 2.2.1. Deep feature extraction

For a L layer deep CNN, a recursive nonlinear activation function ς(.) is used to compute activations $y_{m}^{\left(L\right)}$ produced by the training sample $y_{m}^{\left(0\right)}$. This process can be expressed mathematically as

(2.1) $$y_{m}^{\left(L\right)}=\varsigma \left(\sum_{n=1}^{N^{\left(L-1\right)}}w_{nm}^{\left(L-1\right)}*y_{m}^{\left(L-1\right)}+b_{m}^{\left(L-1\right)}\right),$$

where L is the layer number, $N^{\left(L-1\right)}$ represents the number of kernels at the L−1 layer, m represents the sample id, w is the weight kernel, b is the bias and ‘*’ denotes convolution.

Deep features for each ROI were extracted from the layer that precedes the first fully connected layer. Average pooling (AP) is applied to reduce the deep feature vector length to l. Once all training mass ROIs undergo deep feature extraction, together they form a deep feature dictionary specific to the employed convolutional neural network.

#### 2.2.2. Deep dictionary construction using BlockBoosting

BlockBoost decomposition constructs dictionaries of spatial information from specific regions of the training feature vectors. Each training feature is divided into blocks –that is, spatially localized patches– of length s. Combining all blocks with spatial index i from the training features generates a block-specific dictionary of the form $D^{i}=[d_{1}^{i},d_{2}^{i},\ldots ,d_{k}^{i}]$, where k is the number of training samples. Sparse representations of each block $y_{j}^{i}$ of a test sample $y_{j}$ are computed by optimizing the following objective function given a noise margin ϵ:

(2.2) $${\hat{x}}_{j}^{i}=\text{arg min}\;{\left\|x_{j}^{i}\right\|}_{1}\;\;\text{subject}\;\;\text{to}\;\;{\left\|y_{j}^{i}-D^{i}x_{j}^{i}\right\|}_{2}<\epsilon ,$$

where j represents the test sample id.

**Figure 1.**
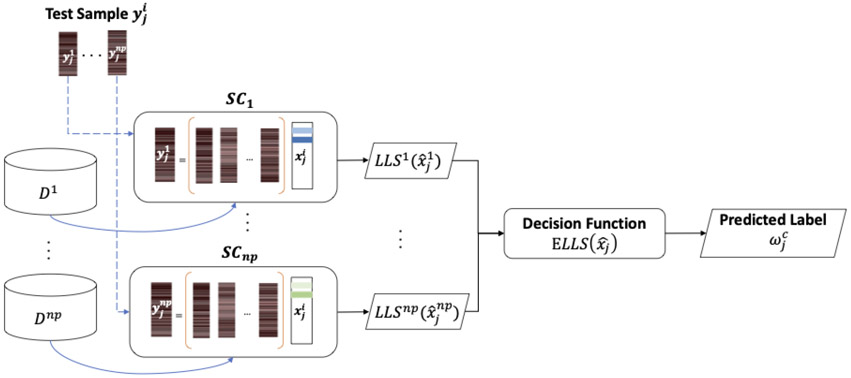
Class prediction flow diagram for BlockBoost decomposition. ${SC}_{i}$ corresponds to the sparse coder specific to block i.

In this manner, sparse representation classification for each test sample is achieved using the corresponding block dictionary from the training set. We denote the number of patches per sample as np=floor(l∕s). Therefore, np unique sparse coders are applied to make a classification decision for a single test sample as shown in Figure 1.

#### 2.2.3. Deep dictionary construction using PatchSampling

PatchSampling decomposition begins in a similar fashion to BlockBoosting by dividing the deep feature vectors into 1-D blocks of length s. The major difference in this decomposition method is that it forms a single deep feature dictionary that is composed from all training patches. A test sample block feature $y_{j}^{i}$ is classified by finding sparse solutions, given the deep features dictionary. A dictionary of this form is not index specific, therefore, it contains many more atoms than the block specific dictionary $D^{i}$. The PatchSampled dictionary is defined as $D=[d_{1}^{1},d_{1}^{2},\ldots ,d_{1}^{np},d_{2}^{1},d_{2}^{2},\ldots ,d_{2}^{np},\ldots ,d_{k}^{1},\ldots d_{k}^{np}]$, where np=floor(l∕s) represents the number of feature patches per subject. The following objective function is optimized to find sparse solutions ${\hat{x}}_{j}^{i}$:

(2.3) $${\hat{x}}_{j}^{i}=\text{arg min}\;{\left\|x_{j}^{i}\right\|}_{1}\;\;\text{subject}\;\;\text{to}\;\;{\left\|y_{j}^{i}-Dx_{j}^{i}\right\|}_{2}<\epsilon .$$

**Figure 2.**
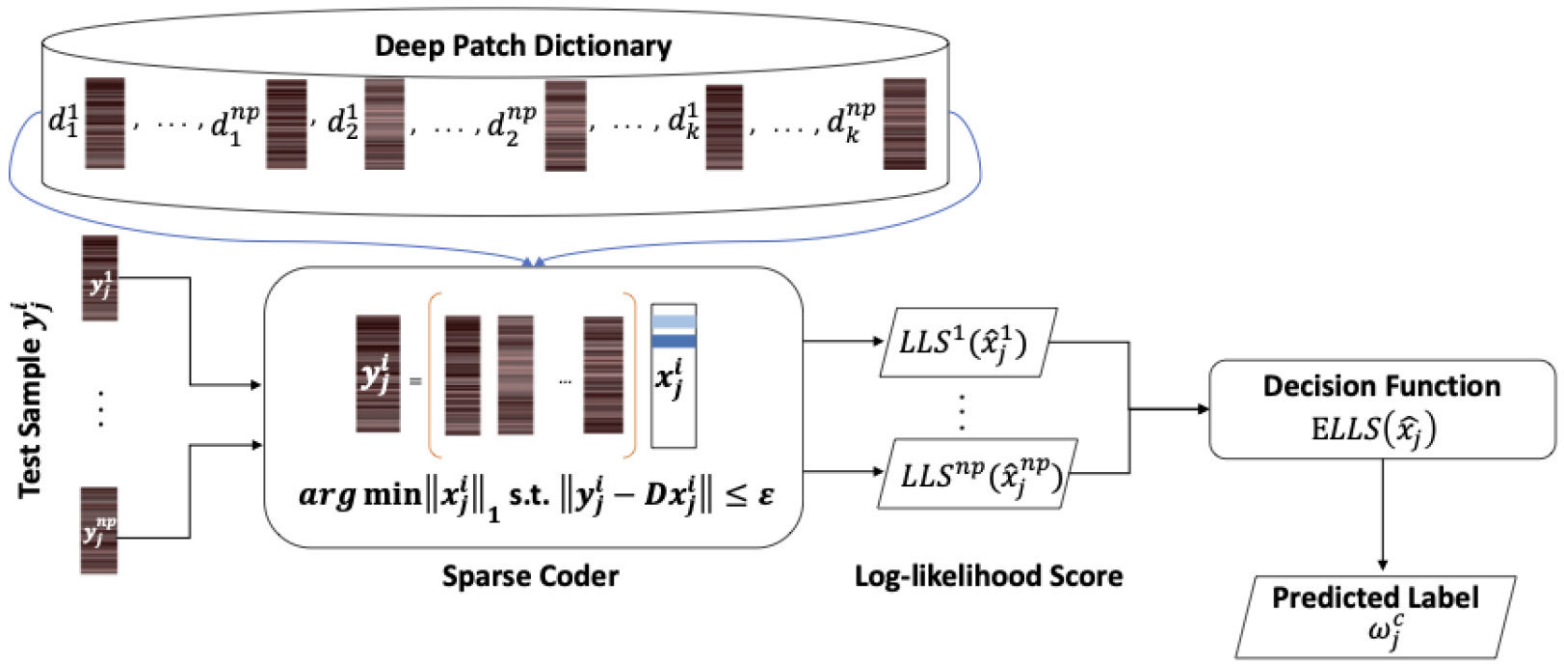
Class prediction flow diagram for PatchSample decomposition.

We illustrate in Figure 2, the class prediction flowchart of a single test sample when PatchSample decomposition is applied. In contrast to BlockBoosting, the PatchSampled deep dictionary D requires just a single sparse coder to approximate each test patch. However, the same number of patch decision scores with BlockBoosting are combined to make a classification decision.

#### 2.2.4. Ensemble classification

Our approach uses a block-based log-likelihood decision score to make an ensemble classification decision. The log-likelihood approximation decision function is defined as,

(2.4) $$LLS\;({\hat{x}}_{j}^{i})=-\log\frac{r_{m}({\hat{x}}_{j}^{i})}{r_{n}({\hat{x}}_{j}^{i})}=-\left[\log\;r_{m}({\hat{x}}_{j}^{i})-\log\;r_{n}({\hat{x}}_{j}^{i})\right],$$

where $r_{m}({\hat{x}}_{j}^{i})$ and $r_{n}({\hat{x}}_{j}^{i})$ are the approximation residuals using the mth and nth class-specific atoms respectively. For example, $r_{m}({\hat{x}}_{j}^{i})={\left\|y_{j}^{i}-D^{i}x_{jm}^{i}\right\|}_{2}$ is the residual of approximation using dictionary atoms and solution coefficients $x_{jm}^{i}$ from the mth class only.

To estimate the ensemble score $ELLS({\hat{x}}_{j})$, we average the individual scores,

(2.5) $$ELLS\;({\hat{x}}_{j})=-\frac{1}{np}\sum_{i}^{np}\left[\log\;r_{m}({\hat{x}}_{j}^{i})-\log\;r_{n}({\hat{x}}_{j}^{i})\right].$$

We employ the sign function to determine the class prediction ${\hat{\omega }}_{c}$, that is

(2.6) $${\hat{\omega }}_{c}=\text{sgn}\left(ELLS\;({\hat{x}}_{j}^{i})\right).$$

## 3. Experiments

We utilized 10-fold cross-validation to evaluate the performance of our DF-SLESA method for classification of breast masses as benign or malignant. For comparison, we performed conventional sparse representation classification (SRC), spatially localized ensemble sparse analysis (SLESA) [34, 35], label-specific dictionary learning SLESA (LS-SLESA) [36] and the aforementioned end-to-end CNNs on the MergedBreast dataset. In conventional SRC experiments, when no block decomposition is applied, we apply dimensionality reduction via principal component analysis (PCA). Table 1 details the deep feature length of the extracted features by convolutional neural network.

### 3.1. Evaluation measures

There are several common performance measures used to assess a classifier, such as precision, recall, accuracy, and area under the ROC curve. In this work, we measure the classification performance by calculating the true positive rate (TPR), true negative rate (TNR), classification accuracy (ACC), and area under the receiver operating characteristic curve (AUC).

Classification accuracy indicates how well a classifier makes a correct prediction. Accuracy is determined using the following equation:

(3.1) $$\text{ACC}=\frac{TP+TN}{TP+TN+FP+FN},$$

where TP, TN, FP, and FN represent the number of true positives, trues negatives, false positives, and false negatives respectively. The true positive rate (TPR) (or recall/sensitivity) is an indication of how well our classifier correctly predicts malignancy,

(3.2) $$\text{TPR}=\frac{TP}{TP+FN}.$$

Similarly, the true negative rate (TNR) indicates how well our classifier correctly classifies benign masses,

(3.3) $$\text{TNR}=\frac{TN}{TN+FP}.$$

The AUC is the area under the receiver operating characteristic (ROC) curve. The ROC curve graphs TPR versus FPR.

### 3.2. Data description

We formed a merged dataset, which we refer to as MergedBreast, by combining benign and malignant masses ROIs from two publicly available datasets, Mammographic Imaging Analysis Society (MIAS) [37, 38] and the Curated Breast Imaging Subset of DDSM (CBIS-DDSM) [39]. The Mammographic Imaging Analysis Society (MIAS) dataset contains 322 MLO view mammograms from a total of 161 patients. The centroid and radius of each mass is provided from radiologist examination readings, and these values are used to generate a bounding box for cropping regions of interest (ROIs). The CBIS-DDSM an updated version of the Digital Database for Screening Mammography (DDSM) dataset that contains 10,239 mammogram images from 1,566 patients. This dataset provides cranial caudal (CC) and MLO mammogram views of breast masses and calcifications with verified pathology. The CBIS-DDSM dataset is considered a standardized version of DDSM as questionable cases were removed, along with other image processing enhancements such as image decompression, noise reduction, and image cropping. The CBIS-DDSM dataset provides ROIs that were cropped by a bounding box about the center of each abnormality. The experimental procedures involving human subjects were approved by the Institutional Review Boards of the institutions, where the data were acquired.

We combined the mass ROIs from the MLO view images from the mass training subset of CBIS-DDSM and ROIs from MIAS to form the MergedBreast dataset. A total of 388 malignant masses and 434 benign masses were considered to form the MergedBreast dataset. The number of malignant and benign samples from both datasets are introduced in Table 2. To prepare the MergedBreast data ROIs for DF-SLESA, SRC and other SLESA methods, the ROIs are downsampled or oversampled to a fixed size.

### 3.3. Pre-processing

To improve the contrast between the masses and their surrounding tissues and reduce the noise level, we applied image enhancement techniques to the complete mammographs and then cropped the mass ROIs. The mammogram image enhancement pipeline begins with median filtering and artifact removal (i.e., removal of label annotations). It then applies unsharp masking, Gaussian filtering and morphological edge enhancement. It finally employs wavelet frames for reconstruction [40], and CLAHE to increase the image contrast.

## 4. Results

**Figure 4.**
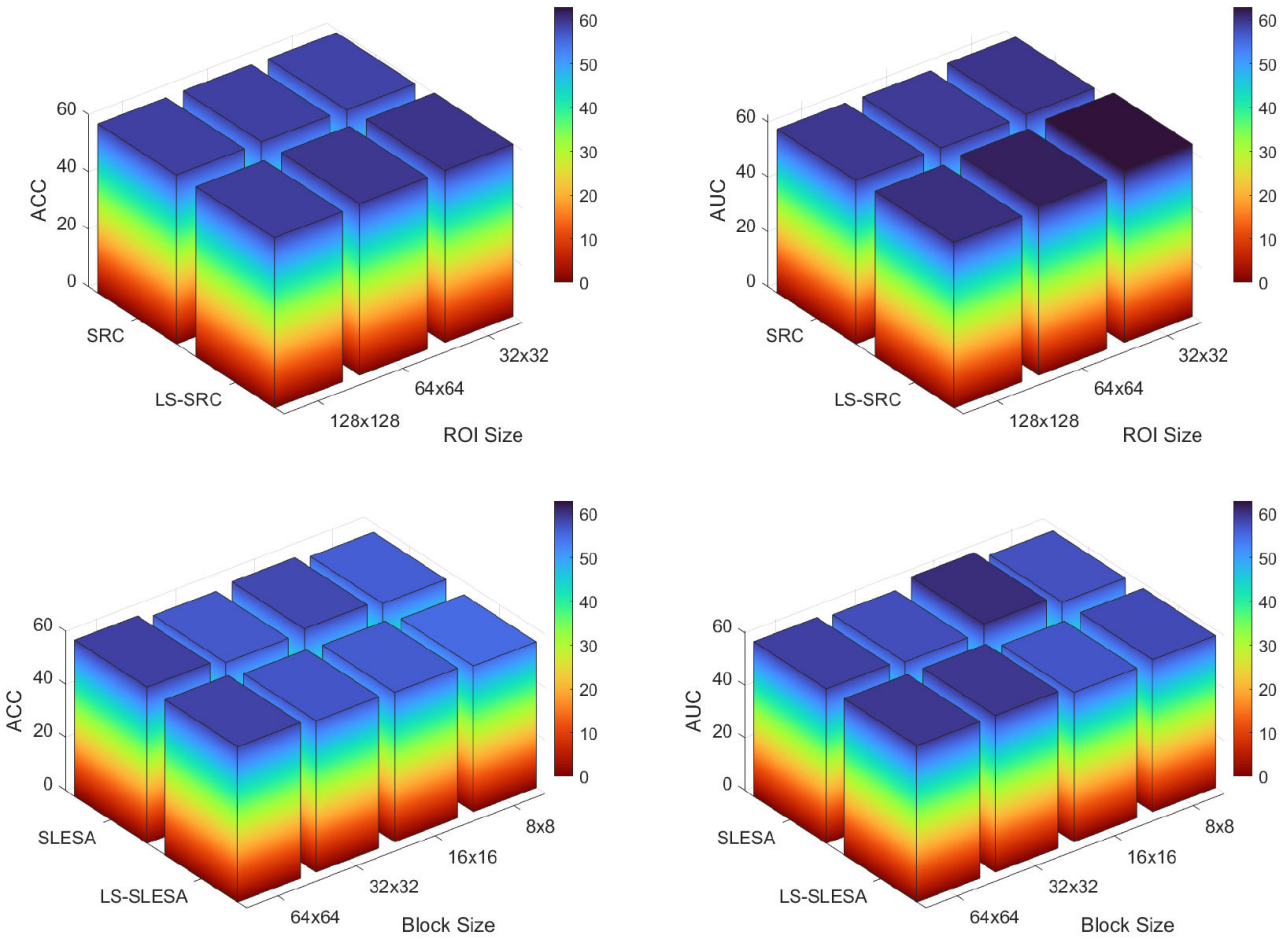
Classification accuracy and area under the ROC curve of SRC and LS-SRC (first row) and SLESA and LS-SLESA (second row).

In Figure 4 (first row) and Table A1 we report the classification performance of conventional SRC and SRC with dictionary learning (LS-SRC) on the MergedBreast dataset. No block decomposition is applied in these experiments, thus providing a baseline for BlockBoosting and PatchSampling performance. We performed resizing to dimensions of 128 × 128, 64 × 64 and 32 × 32 pixels in this set of experiments. We then vectorized the ROIs and applied PCA for dimensionality reduction. The classification rates indicate that dictionary learning and a resizing to dimension of 32 × 32 produces the best classification accuracy. In general, the use of dictionary learning through KSVD [12] slightly improves the ACC and AUC rates.

BlockBoosting and PatchSampling SLESA methods do not improve performance when compared to SRC as shown in Figure 4 (second row) and Table A2. The significant disparity between TPR and TNR is a consistent trend in BlockBoosting and more so in PatchSampling SLESA. Dictionary learning through KSVD improves TPR rates in all PatchSample SLESA experiments, thus providing a better balance between TPR and TNR performances.

As a baseline comparison for our DF-SLESA experiments, we also evaluated the end-to-end classification performances of the same CNNs that we used in DF-SLESA. We applied fine-tuning to networks that were pre-trained on Imagenet. We employed Bayesian optimization to tune the minibatch size (8 to 128) and number of epochs (2 to 80). Geometric transforms, such as rotation and random horizontal and vertical reflection, were used for data augmentation. The network weights were updated in the training stage of CNN classification using Adam optimization with initial learning rate of 10^−3^, learning rate drop factor of 0.95 per epoch, momentum of 0.9 and $\ell_{2}$ regularization of 10^−4^.

**Figure 5.**
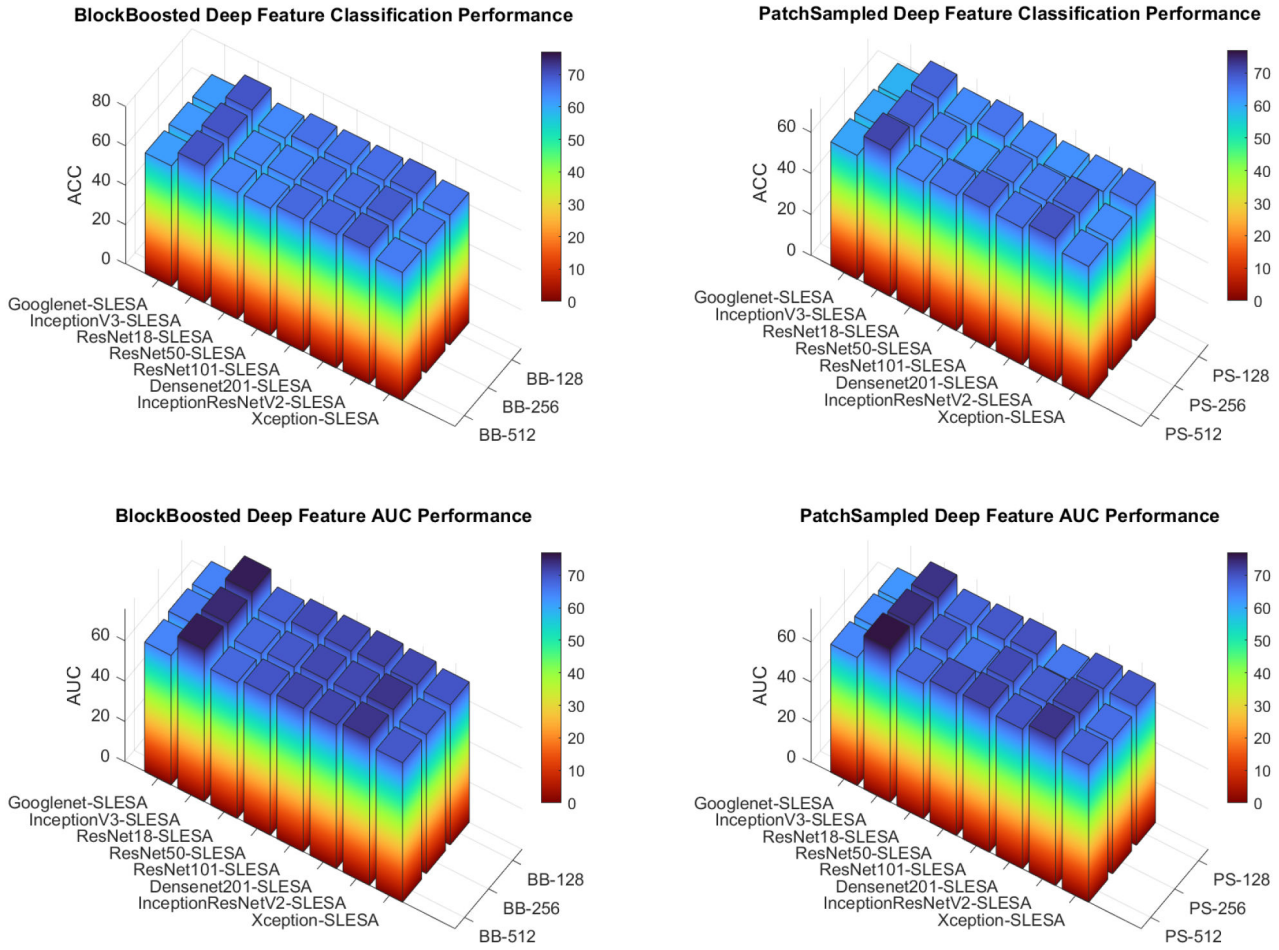
Classification accuracy and area under the ROC curve of DF-SLESA methods using BlockBoosting and PatchSampling.

In our DF-SLESA experiments, we tested block lengths of 512, 256 and 128 in block decomposition. Since ResNet18 deep features have a dimensionality of 512, block decomposition is not possible for block division of block length 512. In Table 3, we report the performance of SRC using deep features. Before block decomposition, we see that deep features improve SRC classification performance significantly, by approximately 10%. Block decomposition further improves classification performance as seen in Tables A3 and A4. We denote the DF-SLESA method by the convolutional neural network name followed by -SLESA. Furthermore, Figure 5 provides a summary of classification accuracy and area under the ROC curve of DF-SLESA methods using BlockBoosting and PatchSampling.

## 5. Discussion

Table 4 provides a comparison summary of the top performances of all methods. Considering the high level of difficulty of benign and malignant mass separation in mammograms, our DF-SLESA methods achieved promising classification performance. The InceptionV3 deep features in conjunction with our SLESA model produce the best performance with the use of block decomposition. InceptionV3-SLESA with PatchSampling with average pooling 256 and block length of 128 produced the best classification performance of 72.31% ACC and 77.04% AUC. InceptionResNetV2-SLESA with BlockBoosting is the second top performing DF-SLESA method. Given that deep features are extracted after 94 convolutional layers in the InceptionV3 network and after 244 convolutional layers in the InceptionResnetV2 network, we note that the number of convolutional layers plays a major role in the quality of the deep features produced.

BlockBoosting and PatchSampling performance in DF-SLESA experiments indicates the limited scalability of sparse classifiers. As the number of training samples increase through block decomposition of smaller lengths, the performance of PatchSampling generally declines. Dimensionality reduction by block decomposition is seemingly most efficient with a reasonable training set size for sparse solvers. While BlockBoosting uses block specific sparse coders and PatchSampling uses a single sparse coder for all test patches, the size of the dictionary seems to have a greater impact on performances than the number of sparse coders used in representation. Convolutional neural networks on the other hand thrive with larger amounts of data, thus having greater scalability.

**Figure 6.**
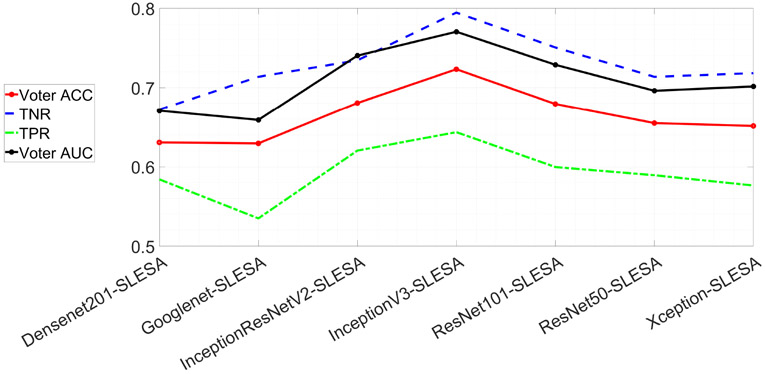
Individual DF-SLESA (BB) performances using a fixed average pooling length of 256 and block length of 128.

Comparing end-to-end CNN classification rates (Table 3) with DF-SLESA classification performance (Figure 5, and Tables A3 and A4) indicates that our sparse ensemble classifier outperforms the neural network classifiers for the majority of CNNs. For instance, SLESA with PatchSample decomposition on InceptionV3 deep features significantly outperforms end-to-end classification using InceptionV3 solely. Densenet201 end-to-end classification produced the top accuracy performance among end-to-end CNN classifiers, achieving an ACC of 72.29% and AUC of 77.51%. However, DF-SLESA produces competitive rates with CNNs. DF-SLESA methods outperform CNN classification for Googlenet, InceptionV3, ResNet50, ResNet101 and InceptionResNetV2 features. Overall, the use of block decomposition yields improvement in DF-SLESA performance for most network deep features, as it reduces the dimensionality of the approximation problem. In addition, the InceptionV3-SLESA method achieved the highest ACC of 72.31% and second best AUC of 77.04%. Figure 6 displays the ACC, AUC, TPR and TNR measures produced by DF-SLESA with average pooling of 256 and BlockBoosting with block length 128 corresponding to the top InceptionV3-SLESA performer.

Furthermore, comparisons with related work may not be straightforward as the reported results of mammography classification seem to be highly heterogeneous. The major factors that may cause the variability of results are the type of mammograms (digital or film screen) and views (MLO and/or CC), the classification task (normal versus abnormal tissue, benign versus malignant, masses and/or calcifications), and the use of ROIs versus complete mammograms [2,21,22]. In a directly comparable work published in [41], the authors employ an ad hoc CNN architecture for classification of benign and malignant masses from the CBIS-DDSM dataset and achieved 71.19% ACC. In [42], the authors performed a similar task of benign versus malignant mass classification using sparse representation classification on the DDSM dataset. Their patch ROI characterization and classification strategy was assessed on 80 masses with various mass margins and obtained 70.00% classification accuracy.

### System interpretability.

The subject of system interpretability becomes increasingly important as machine learning is employed in multiple domains [43]. In clinical applications, interpretability may help to increase the level of confidence in the applicability of a system. A strength of sparse approximation-based approaches is their interpretability as it is possible to monitor the sparse solutions, the approximations, and the residuals.

**Figure 7.**
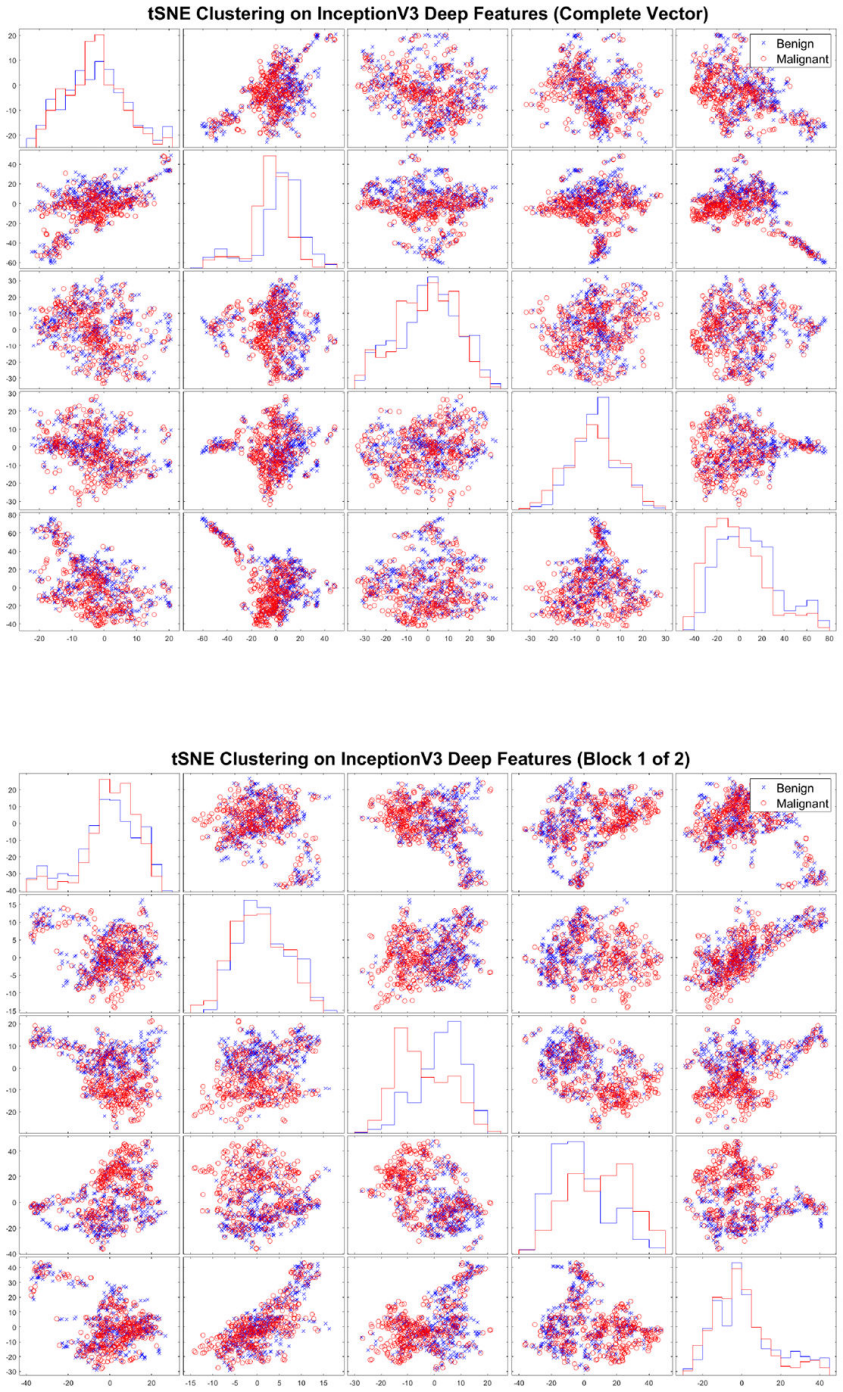
t-SNE clustering plots with 5-D embedding of InceptionV3 deep features produced by DF-SRC (top) and DF-SLESA with BlockBoosting (bottom). The greatest KL divergence measure for DF-SRC, 0.3304, is produced by component two. The greatest KL divergence measure for DF-SLESA(BB), 0.1843, is produced by component two.

We first aim to obtain insight into the deep dictionaries that we use for sparse approximations. In Figure 7, we display t-SNE clustering plots using 5-D embeddings of InceptionV3 deep feature data. We observe, through the single feature histograms and pair-wise scatterplots, significant similarity of benign and malignant deep features for the complete feature vector and for the first block features. However, some feature components show greater separability, such as the second component of the complete InceptionV3 feature data. We computed the Kullback-Leibler (KL) divergence to measure the class discrimination for each feature component. The greatest KL divergence measure for the complete feature vector data, 0.3304, is produced by component two. The greatest KL divergence measure for the first block feature data, 0.1843, is produced by component two.

**Figure 8.**
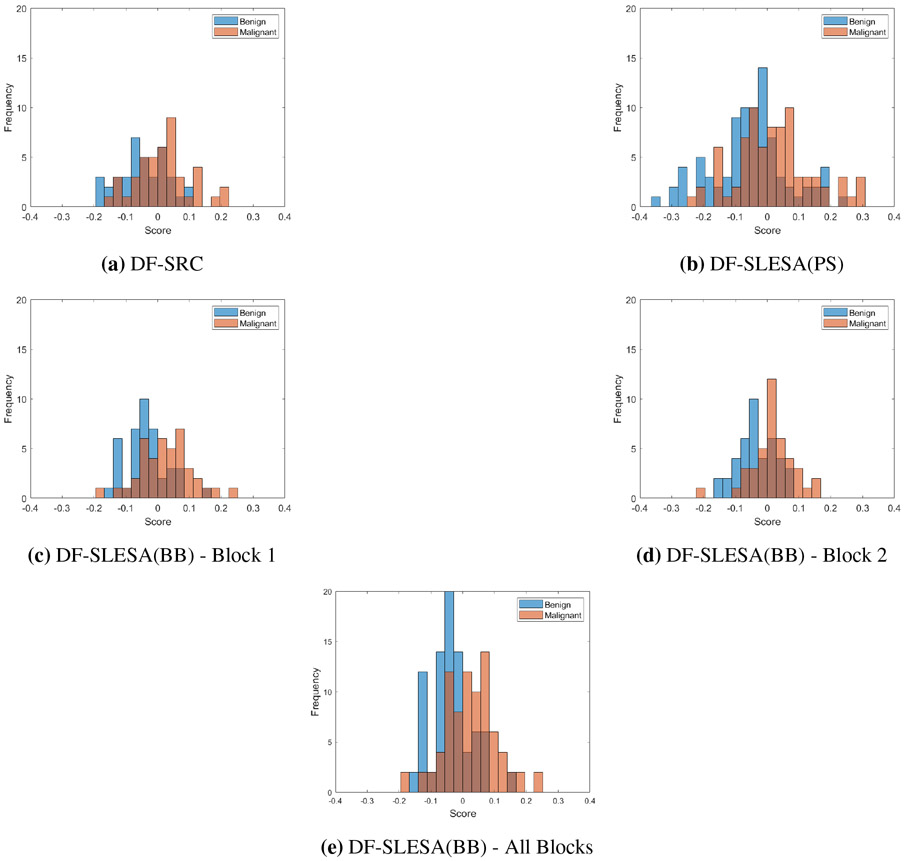
Decision scores for a single test fold in 10 CV by class using InceptionV3 deep features using (a) no block decomposition, (b) PatchSampling with block length of 512, (c)–(d) BlockBoosting with a block length of 512, (e) combined scores for both blocks for BlockBoosting with a block length of 512.

Next, we explore the decision function of DF-SLESA techniques defined in (2.4) that uses log ratios of residuals by displaying histograms of the decision scores. We calculated the histogram of scores for a single test fold in 10-fold cross-validation. We used InceptionV3-SLESA to explore the cases of a single block, PatchSampling, and BlockBoosting. Figure 8 displays the histograms of decision scores for each case. We observe that the scores approximately form two distributions that correspond to each mass type. We note that the scores of malignant masses are more dispersed than those of the benign masses. This explains the lower true positive rate in our results. In addition, the probability of error generally decreases as the score magnitude increases. This indicates that *ELLS* values could be used to produce heat maps, or to provide a level of confidence that could help to make a decision in a diagnostic workflow.

## 6. Conclusions

Benign and malignant mass separation is considered a more challenging task in machine learning than natural image classification. In this work, we combine the inductive representation capacity of CNNs to form dictionaries for sparse approximation-based classification of mass ROIs in a merged mammography dataset. Our aim is to show that this approach yields an effective and interpretable classification technique. Our results indicate that deep features produce numerically feasible sparse approximations, which ultimately improves the performance of our sparse analysis methods. The proposed DF-SLESA approach produces competitive results with contemporary machine learning and deep learning techniques of the literature.

## Figures and Tables

**Figure 3.**
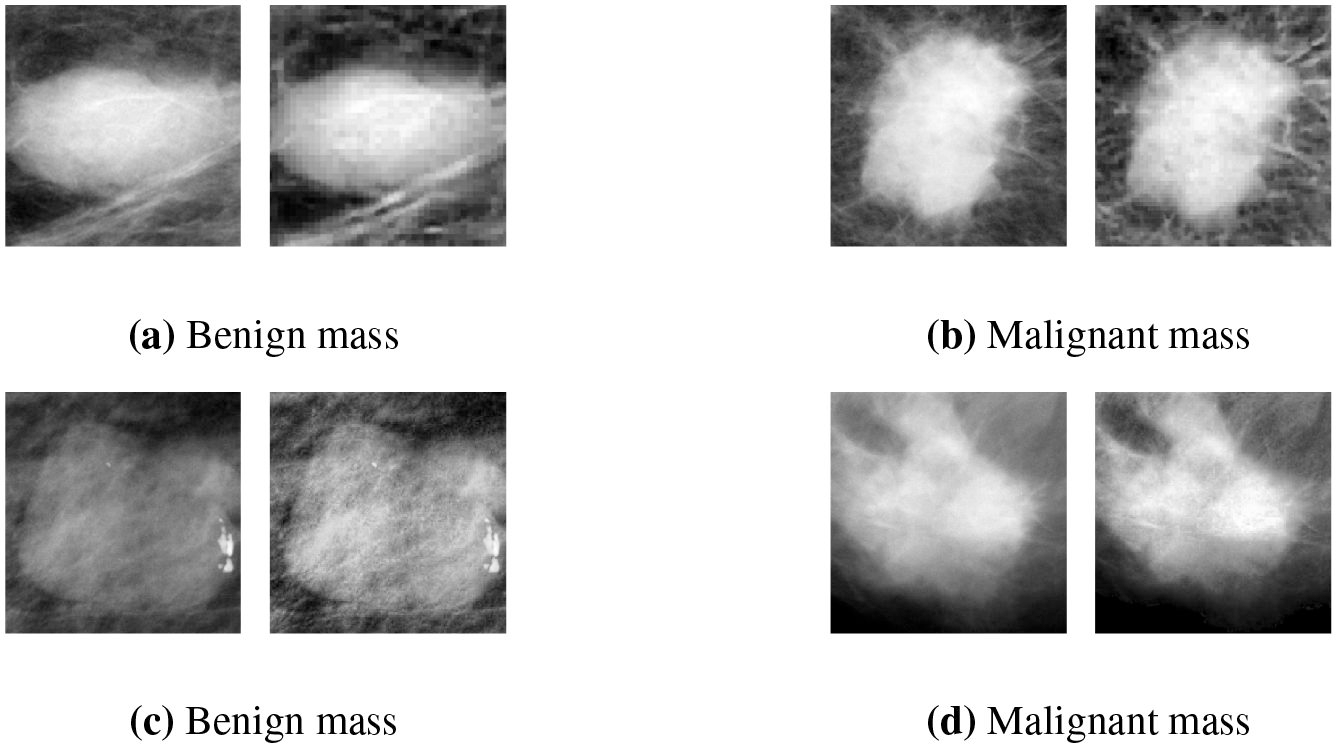
Examples of pre-processing enhancement on benign and malignant mass ROIs. (a) a benign mass ROI before and after enhancement (left-to-right) from the MIAS dataset, (b) a malignant mass ROI before and after enhancement (left-to-right) from the MIAS dataset, (c) a benign mass ROI before and after enhancement (left-to-right) from the CBIS-DDSM dataset, (d) a malignant mass ROI before and after enhancement (left-to-right) from the CBIS-DDSM dataset.

**Table 1.** Dimensionality of extracted deep features by convolutional neural network.

Neural network	Deep feature length (l)
Googlenet	832
InceptionV3	1280
ResNet18	512
ResNet50	2048
ResNet101	2048
Densenet201	1920
InceptionResNetV2	1536
Xception	2048

**Table 2.** Number of malignant and benign samples from both datasets.

Dataset	Number of Malignant Masses	Number of Benign Masses	Total
CBIS-DDSM	341	370	711
MIAS	47	64	111
Total	388	434	822

**Table 3.** Convolutional neural network and deep feature SRC classification performance.

Method	TPR (%)	TNR (%)	ACC (%)	AUC (%)
End-to-End CNN				
Googlenet	68.81	52.06	60.44	64.55
InceptionV3	56.19	73.71	64.95	68.96
ResNet18	59.28	64.18	61.73	65.14
ResNet50	53.09	72.94	63.02	67.82
ResNet101	56.44	79.64	68.04	72.05
Densenet201	62.37	82.22	**72.29**	**77.51**
InceptionResNetV2	60.31	65.98	63.14	67.03
Xception	54.90	82.73	68.81	72.69
DF-SRC				
Googlenet-SRC	61.24	61.89	61.59	63.93
InceptionV3-SRC	66.41	72.52	69.63	75.23
ResNet18-SRC	67.47	67.21	64.27	67.47
ResNet50-SRC	62.27	68.13	65.37	69.16
ResNet101-SRC	62.79	73.21	68.29	72.45
Densenet201-SRC	65.12	71.13	68.29	72.30
InceptionResNetV2-SRC	67.70	72.75	**70.37**	**72.58**
Xception-SRC	59.43	70.67	65.37	67.67

**Table 4.** Comparison summary of top performances of all methods.

Method	TPR (%)	TNR (%)	ACC (%)	AUC (%)
SRC (64)	68.48	50.58	59.02	59.53
LS-SRC (32)	70.54	50.58	60.00	62.78
SLESA (BB-64)	46.51	70.00	58.90	58.93
LS-SLESA (BB-64)	51.68	64.90	58.66	59.69
CNN (Densenet201)	62.37	82.22	**72.29**	**77.51**
DF-SRC	67.70	72.75	70.37	72.58
InceptionV3-SLESA (PS-512)	65.12	77.14	**71.46**	**76.46**
InceptionV3-SLESA (AP-256, BB-128)	64.34	79.45	**72.31**	**77.04**

* Notes: BB-# : BlockBoosting with block size #; PS-# : PatchSampling with block size #; AP-# : average pooling with block size #

## Appendix

Appendix tables that list numeric values of performance measures from the experiments of Sections 4 and 5. These results support our research findings.

- Table A1: performance of SRC and LS-SRC using ROI resizing of 128 × 128, 64 × 64, and 32 × 32.
- Table A2: performance of SLESA and LS-SLESA with BlockBoosting and PatchSampling using block sizes of 64 × 64, 32 × 32, 16 × 16, and 8 × 8.
- Table A3: performance of DF-SLESA with BlockBoosting using block lengths of 512, 256, 128

- Table A4: performance of DF-SLESA with PatchSampling using block lengths of 512, 256, 128

**Table A1.** Performance of conventional sparse representation classification (SRC), and SRC with dictionary learning (LS-SRC).

Method	ROI Size	TPR (%)	TNR (%)	ACC (%)	AUC (%)
SRC	128 × 128	71.06	48.04	58.90	59.69
SRC	64 × 64	68.48	50.58	**59.02**	59.53
SRC	32 × 32	70.54	58.27	58.78	**59.93**
LS-SRC	128 × 128	68.22	51.27	59.27	60.36
LS-SRC	64 × 64	68.99	51.50	59.76	61.57
LS-SRC	32 × 32	70.54	50.58	**60.00**	**62.78**

**Table A2.** Breast mass classification using SLESA and LS-SLESA by BlockBoosting and PatchSampling (ROI Size: 128 × 128).

BlockBoosting					
Method	Block Size	TPR (%)	TNR (%)	ACC (%)	AUC (%)
SLESA	64 × 64	46.51	70.00	**58.90**	**58.93**
SLESA	32 × 32	39.53	72.29	56.83	57.70
SLESA	16 × 16	49.10	66.28	58.17	60.64
SLESA	8 × 8	29.72	80.00	56.34	57.47
LS-SLESA	64 × 64	51.68	64.90	**58.66**	**59.69**
LS-SLESA	32 × 32	45.74	67.21	57.07	59.74
LS-SLESA	16 × 16	42.89	68.59	56.46	57.04
LS-SLESA	8 × 8	27.65	79.45	55.00	57.99
PatchSampling					
Method	Block Size	TPR (%)	TNR (%)	ACC (%)	AUC (%)
SLESA	64 × 64	38.50	72.58	**56.52**	55.78
SLESA	32 × 32	10.05	91.01	52.80	54.65
SLESA	16 × 16	10.31	96.08	55.60	**56.47**
SLESA	8 × 8	2.84	97.93	53.04	55.25
LS-SLESA	64 × 64	49.35	60.37	55.18	57.51
LS-SLESA	32 × 32	46.13	61.98	54.50	54.67
LS-SLESA	16 × 16	42.01	71.20	**57.42**	**58.12**
LS-SLESA	8 × 8	28.35	80.65	55.96	55.03

**Table A3.** BlockBoosted deep feature classification.

Method	DF Length	Block Length	TPR (%)	TNR (%)	ACC (%)	AUC (%)
(Block Length 512)						
Googlenet-SLESA	832	512	60.98	61.66	61.34	64.32
InceptionV3-SLESA	1280	512	65.89	73.21	**69.76**	75.63
ResNet50-SLESA	2048	512	61.50	67.44	64.63	69.59
ResNet101-SLESA	2048	512	61.50	72.75	67.44	71.31
Densenet201-SLESA	1920	512	64.34	70.90	67.81	71.37
InceptionResNetV2-SLESA	1536	512	65.89	72.52	69.39	73.29
Xception-SLESA	2048	512	62.53	67.44	65.12	69.19
(Block Length 256)						
Googlenet-SLESA	832	256	60.21	63.05	61.71	65.42
InceptionV3-SLESA	1280	256	66.15	72.75	69.63	74.49
ResNet18-SLESA	512	256	59.43	67.90	63.90	67.64
ResNet50-SLESA	2048	256	60.98	68.36	64.88	69.28
ResNet101-SLESA	2048	256	62.02	71.82	67.20	71.09
Densenet201-SLESA	1920	256	64.86	69.28	67.20	71.05
InceptionResNetV2-SLESA	1536	256	65.12	72.29	68.90	73.10
Xception-SLESA	2048	256	57.62	72.75	65.61	69.12
(Block Length 128)						
Googlenet-SLESA	832	128	60.21	62.82	61.59	64.66
InceptionV3-SLESA	1280	128	66.41	72.52	69.63	**75.67**
ResNet18-SLESA	512	128	59.17	67.67	63.66	68.54
ResNet50-SLESA	2048	128	59.17	73.21	66.59	70.96
ResNet101-SLESA	2048	128	61.24	71.59	66.71	71.28
Densenet201-SLESA	1920	128	61.76	71.82	67.07	71.86
InceptionResNetV2-SLESA	1536	128	62.79	73.67	68.54	71.27
Xception-SLESA	2048	128	56.85	74.13	65.98	69.54

**Table A4.** PatchSampled deep feature classification.

Method	DF Length	Patch Length	TPR (%)	TNR (%)	ACC (%)	AUC (%)
(Patch Length 512)						
Googlenet-SLESA	832	512	60.21	60.51	60.37	63.82
InceptionV3-SLESA	1280	512	65.12	77.14	**71.46**	**76.46**
ResNet50-SLESA	2048	512	57.88	73.04	65.90	70.68
ResNet101-SLESA	2048	512	60.47	75.81	68.57	71.83
Densenet201-SLESA	1920	512	62.02	70.90	66.71	69.64
InceptionResNetV2-SLESA	1536	512	65.63	73.67	69.88	73.18
Xception-SLESA	2048	512	57.62	70.97	64.68	68.49
(Patch Length 256)						
Googlenet-SLESA	832	256	55.30	64.90	60.37	63.49
InceptionV3-SLESA	1280	256	53.09	82.03	68.37	74.18
ResNet18-SLESA	512	256	61.76	69.52	65.85	69.96
ResNet50-SLESA	2048	256	50.52	73.44	62.61	66.75
ResNet101-SLESA	2048	256	60.05	71.82	66.26	70.71
Densenet201-SLESA	1920	256	61.76	69.52	65.85	69.14
InceptionResNetV2-SLESA	1536	256	62.02	73.50	68.09	71.76
Xception-SLESA	2048	256	60.05	66.05	63.22	66.87
(Patch Length 128)						
Googlenet-SLESA	832	128	53.75	63.13	58.71	62.19
InceptionV3-SLESA	1280	128	61.60	74.19	68.25	73.52
ResNet18-SLESA	512	128	57.88	68.66	63.58	68.27
ResNet50-SLESA	2048	128	60.05	71.43	66.06	69.20
ResNet101-SLESA	2048	128	57.47	70.28	64.23	70.24
Densenet201-SLESA	1920	128	54.90	69.82	62.78	65.62
InceptionResNetV2-SLESA	1536	128	65.72	62.90	64.23	69.63
Xception-SLESA	2048	128	55.41	75.58	66.06	69.34

## References

[1] Nagai H, Kim YH, Cancer prevention from the perspective of global cancer burden patterns, J. Thorac. Dis, 9 (2017), 448–451. 10.21037/jtd.2017.02.75 28449441 PMC5394024

[2] Yu X, Zhou Q, Wang S, Zhang YD, A systematic survey of deep learning in breast cancer, Int. J. Intell. Syst, 37 (2022), 152–216. 10.1002/int.22622

[3] Chhikara BS, Parang K, Global cancer statistics 2022: the trends projection analysis, Chem. Biol. Lett, 10 (2023), 451.

[4] Waks AG, Winer EP, Breast cancer treatment: a review, Jama, 321 (2019), 288–300. 30667505 10.1001/jama.2018.19323

[5] Watkins EJ, Overview of breast cancer, J. Am. Acad. PAs, 32 (2019), 13–17. 10.1097/01.JAA.0000580524.95733.3d 31513033

[6] Elmore JG, Jackson SL, Abraham L, Miglioretti DL, Carney PA, Geller BM, , Variability in interpretive performance at screening mammography and radiologists characteristics associated with accuracy, Radiology, 253 (2009), 641–651. 10.1148/radiol.2533082308 19864507 PMC2786197

[7] Caballo M, Hernandez AM, Lyu SH, Teuwen J, Mann RM, Van Ginneken B, , Computer-aided diagnosis of masses in breast computed tomography imaging: deep learning model with combined handcrafted and convolutional radiomic features, J. Med. Imaging, 8 (2021), 024501. 10.1117/1.JMI.8.2.024501 PMC8005916 33796604

[8] Eltoukhy MM, Faye I, Samir BB, A statistical based feature extraction method for breast cancer diagnosis in digital mammogram using multiresolution representation, Comput. Biol. Med, 42 (2012), 123–128. 10.1016/j.compbiomed.2011.10.016 22115076

[9] Khan S, Yong SP, A comparison of deep learning and hand crafted features in medical image modality classification, in 2016 3rd International Conference on Computer and Information Sciences (ICCOINS), (2016), 633–638. 10.1109/ICCOINS.2016.7783289

[10] Ke L, Mu N, Kang Y, Mass computer-aided diagnosis method in mammogram based on texture features, in 2010 3rd International Conference on Biomedical Engineering and Informatics, (2010), 354–357. 10.1109/BMEI.2010.5639515

[11] Donoho DL, Elad M, Optimally sparse representation in general (nonorthogonal) dictionaries via l1 minimization, Proc. Natl. Acad. Sci, 100 (2003), 2197–2202. 10.1073/pnas.0437847100 16576749 PMC153464

[12] Aharon M, Elad M, Bruckstein A, K-svd: An algorithm for designing overcomplete dictionaries for sparse representation, IEEE Trans. Signal. Process, 54 (2006), 4311–4322. 10.1109/TSP.2006.881199

[13] Wright J, Ma Y, Mairal J, Sapiro G, Huang TS, Yan S, Sparse representation for computer vision and pattern recognition, Proc. IEEE, 98 (2010), 1031–1044. 10.1109/JPROC.2010.2044470

[14] Plenge E, Klein SS, Niessen WJ, Meijering E, Multiple sparse representations classification, PLos ONE. 10.1371/journal.pone.0131968 PMC4503426 26177106

[15] Kohli MD, Summers RM, Geis JR, Medical image data and datasets in the era of machine learning whitepaper from the 2016 c-mimi meeting dataset session, J. Digit. Imaging, 30 (2017), 392–399. 10.1007/s10278-017-9976-3 28516233 PMC5537092

[16] Kim HE, Cosa-Linan A, Santhanam N, Jannesari M, Maros ME, Ganslandt T, Transfer learning for medical image classification: A literature review, BMC Med. Imaging, 22 (2022), 69. 10.1186/s12880-022-00793-7 35418051 PMC9007400

[17] Alzubaidi L, Al-Amidie M, Al-Asadi A, Humaidi AJ, Al-Shamma O, Fadhel MA, , Novel transfer learning approach for medical imaging with limited labeled data, Cancers, 13 (2021), 1590. 10.3390/cancers13071590 33808207 PMC8036379

[18] Lévy D, Jain A, Breast mass classification from mammograms using deep convolutional neural networks, preprint, arXiv:1612.00542.

[19] Huynh BQ, Li H, Giger ML, Digital mammographic tumor classification using transfer learning from deep convolutional neural networks, J. Med. Imaging, 3 (2016), 034501. 10.1117/1.JMI.3.3.034501 PMC4992049 27610399

[20] Jiang F, Liu H, Yu S, Xie Y, Breast mass lesion classification in mammograms by transfer learning, in Proceedings of the 5th International Conference on Bioinformatics and Computational Biology, (2017), 59–62. 10.1145/3035012.3035022

[21] Burt JR, Torosdagli N, Khosravan N, RaviPrakash H, Mortazi A, Tissavirasingham F, , Deep learning beyond cats and dogs: recent advances in diagnosing breast cancer with deep neural networks, Br. J. Radiol, 91 (2018), 20170545. 10.1259/bjr.20170545 29565644 PMC6223155

[22] Munir K, Elahi H, Ayub A, Frezza F, Rizzi A, Cancer diagnosis using deep learning: A bibliographic review, Cancers, 11 (2019), 1235. 10.3390/cancers11091235 31450799 PMC6770116

[23] Al-Antari MA, Han SM, Kim TS, Evaluation of deep learning detection and classification towards computer-aided diagnosis of breast lesions in digital x-ray mammograms, Comput. Methods Programs Biomed, 196 (2020), 105584. 10.1016/j.cmpb.2020.105584 32554139

[24] Wu N, Phang J, Park J, Shen Y, Huang Z, Zorin M, , Deep neural networks improve radiologists’ performance in breast cancer screening, IEEE Trans. Med. Imaging, 39 (2020), 1184–1194. 10.1109/TMI.2019.2945514 31603772 PMC7427471

[25] Saber A, Sakr M, Abo-Seida OM, Keshk A, Chen H, A novel deep-learning model for automatic detection and classification of breast cancer using the transfer-learning technique, IEEE Access, 9 (2021), 71194–71209. 10.1109/ACCESS.2021.3079204

[26] Shen Y, Wu N, Phang J, Park J, Liu K, Tyagi S, , An interpretable classifier for high-resolution breast cancer screening images utilizing weakly supervised localization, Med. Image Anal, 68 (2021), 101908. 10.1016/j.media.2020.101908 33383334 PMC7828643

[27] Szegedy C, Liu W, Jia Y, Sermanet P, Reed S, Anguelov D, , Going deeper with convolutions, in Proceedings of the IEEE conference on computer vision and pattern recognition, (2015), 1–9. 10.1109/CVPR.2015.7298594

[28] Szegedy C, Vanhoucke V, Ioffe S, Shlens J, Wojna Z, Rethinking the inception architecture for computer vision, in Proceedings of the IEEE conference on computer vision and pattern recognition, (2016), 2818–2826. 10.1109/CVPR.2016.308

[29] He K, Zhang X, Ren S, Sun J, Deep residual learning for image recognition, in Proceedings of the IEEE conference on computer vision and pattern recognition, (2016), 770–778. 10.1109/CVPR.2016.90

[30] Huang G, Liu Z, Van Der Maaten L, Weinberger KQ, Densely connected convolutional networks, in Proceedings of the IEEE conference on computer vision and pattern recognition, (2017), 4700–4708. 10.1109/CVPR.2017.243

[31] Szegedy C, Ioffe S, Vanhoucke V, Alemi A, Inception-v4, Inception-ResNet and the impact of residual connections on learning, in Proceedings of the AAAI conference on artificial intelligence, 31 (2017). 10.1609/aaai.v31i1.11231

[32] Chollet F, Xception: Deep learning with depthwise separable convolutions, in Proceedings of the IEEE conference on computer vision and pattern recognition, (2017), 1251–1258. 10.1109/CVPR.2017.195

[33] Harris CE, Makrogiannis S, Breast mass characterization using sparse approximations of patch-sampled deep features, in Medical Imaging 2023: Computer-Aided Diagnosis, 2023.

[34] Zheng K, Harris C, Bakic P, Makrogiannis S, Spatially localized sparse representations for breast lesion characterization, Comput. Biol. Med, 123 (2020), 103914. 10.1016/j.compbiomed.2020.103914 32768050 PMC7416513

[35] Zheng K, Harris CE, Jennane R, Makrogiannis S, Integrative blockwise sparse analysis for tissue characterization and classification, Artif. Intell. Med, 107 (2020), 101885. 10.1016/j.artmed.2020.101885 32828443 PMC7445355

[36] Makrogiannis S, Zheng K, Harris C, Discriminative localized sparse approximations for mass characterization in mammograms, Front. Oncol, 11 (2021), 725320. 10.3389/fonc.2021.725320 35036353 PMC8755640

[37] Oliver A, Freixenet J, Marti J, Perez E, Pont J, Denton ER, , A review of automatic mass detection and segmentation in mammographic images, Med. Image Anal, 14 (2010), 87–110. 10.1016/j.media.2009.12.005 20071209

[38] Matheus BRN, Schiabel H, Online mammographic images database for development and comparison of cad schemes, J. Digit. Imaging, 24 (2011), 500–506. 10.1007/s10278-010-9297-2 20480383 PMC3092049

[39] Lee RS, Gimenez F, Hoogi A, Miyake KK, Gorovoy M, Rubin DL, A curated mammography data set for use in computer-aided detection and diagnosis research, Sci. Data, 4 (2017), 1–9. 10.1038/sdata.2017.177 PMC5735920 29257132

[40] Do MN, Vetterli M, Framing pyramids, IEEE Trans. Signal Proc, 51 (2003), 2329–2342. 10.1109/TSP.2003.815389

[41] Duggento A, Aiello M, Cavaliere C, Cascella GL, Cascella D, Conte G, , An ad hoc random initialization deep neural network architecture for discriminating malignant breast cancer lesions in mammographic images, Contrast Media Mol. Imaging, 2019 (2019), 5982834. 10.1155/2019/5982834 31249497 PMC6556299

[42] Narvaez F, Rueda A, Romero E, Breast masses classification using a sparse representation, in Proceedings of the 2nd International Workshop on Medical Image Analysis and Description for Diagnosis Systems, (2011), 26–33.

[43] Linardatos P, Papastefanopoulos V, Kotsiantis S, Explainable AI: A review of machine learning interpretability methods, Entropy, 23 (2020), 18. 10.3390/e23010018 33375658 PMC7824368

